# Illegal drugs sensor: Performance evaluation and identification based on terahertz photonic crystal fiber

**DOI:** 10.1371/journal.pone.0327013

**Published:** 2025-06-27

**Authors:** Kayab Khandakar, Jabin Tasnin Upoma, Taib Hasan, A. H. M. Iftekharul Ferdous, Diponkar Kundu, Md. Omar Faruk, Md. Feroz Ali, Md. Shahorin Islam Shaun

**Affiliations:** Department of Electrical and Electronic Engineering, Pabna University of Science and Technology, Pabna, Bangladesh; Universiti Brunei Darussalam, BRUNEI DARUSSALAM

## Abstract

Excessive hormone release, the possibility of sleep disturbances, and a brief and quick improvement in the functioning of many organs, the physiological system, the nerves, etc. are all consequences of the abuse of incentive medications. Illegal narcotics have terrible long-term impacts on human health, including the possibility of death, in addition to their immediate effects. These consequences highlight the need for more obviousness and accuracy in the detection of illicit drugs, as well as for their detection to be done gently, effectively, and consistently. This work introduces an illicit drug sensor based on PCF, with an eye toward these as the primary targets. Three illegal drugs – ketamine, amphetamine, and cocaine – have been simulated for the sensor. Two types of circular air holes in cladding of varying sizes have been developed for a single core PCF. The cladding has three-layer chain and wind turbine-shaped air holes, and a circular air hole in the core region that will be used to field test drug samples, all included to achieve low confinement losses and high sensitivity. A maximum Relative Sensitivity (RS) of 99.92%, 99.12% and 98.83% at ketamine, amphetamine, and cocaine respectively is revealed by the recently established PCF analysis, which was presented out right away. Furthermore, we looked at the Confinement Loss (CL) associated with these illicit drugs, which was around 1.275 × 10^−7^ dB/m, 2.653 × 10^−9^ dB/m, and 4.106 × 10^−10^ dB/m, besides Effective Material Loss (EML) of 0.0042 cm^-1^, 0.0044 cm^-1^ and 0.0045 cm^-1^. Refractive index changes in PCF are usually the cause of action for PCF-based biosensors. These modifications have an impact on how light travels within the fiber. Drug molecules interact with light as a result of changes in the optical properties of the core that occur during light propagation through it.

## 1. Introduction

Drugs that are prohibited from being owned or used by anybody by law are known as illegal drugs. Drugs that are illegal are the deadliest and addictive. The primary categories of illegal drugs are hallucinogens, barbiturates, cannabinoids, benzodiazepines, opioids and alcohol. Three commonly utilized illegal substances include cocaine, amphetamine, and ketamine [1]. The four main groups of illegal substances can be distinguished based on their main effects: stimulants, opium-based painkillers, nervines like alcohol and hallucinogenic drugs. People who use various illegal drugs experience different affects and these effects are influenced by a multitude of conditions. These variables include the kind of medicine, the dosage used, individual traits including body type and health vulnerabilities, and more [2]. Antidepressants like ketamine have hypnotic, short-term memory loss, and pain-relieving properties. When ketamine is misused, mental and emotional issues arise [3]. Psychotic sickness is caused by amphetamine intoxication. The combination of amphetamines has firmly established itself as a popular and widespread drug exploitation method. Although amphetamine and cocaine have different structural makeups, they have similar clinical and neurochemical effects [4]. Conventional methods of testing for illegal drugs entail analyzing a biological sample. Testing for the availability of cocaine, amphetamine, and ketamine in saliva and urine is done, but occasionally the results are not ideal. Blood testing is more reliable and should only be done in extreme cases [5]. With the help of this proposed study, a biosensor based on PCF becomes a more advanced, precise, and effective way to test blood for illicit drugs [6]. Since PCF has several remarkable advantages over ordinary fiber, PCF is a godsend for fiber-optic communication nowadays. As a result, there is growth in communication sectors and various sensing technologies [7–14]. Real-world applications have been using PCFs since 1996 [15], which are identified by their transverse periodic microstructure. HC-PCF, SC-PCF and porous core PCF are three main classes of PCFs that are distinguished by their structural architecture. In communication applications, each category can be used to transmit data more quickly and securely. Because they are incapable of injecting the analyte into the fiber, SC-PCFs are not appropriate for use in spectroscopy or sensing applications [16]. In addition, PCFs have been distinguished with solid and hollow cores; sensing capability of solid core PCF is lower than that of the hollow-core (HCF) one. They achieve a lot of interaction with light and the analyte in its core which makes them very good for detection liquids or low refractive index (RI) gaseous subject. Larger core size is a possible means to improve sensitivity of the sensor by increasing overlap between mode fields, but must also carefully consider PCF fabrication difficulty. Analytes sensing by PCF is the subject of many more studies these days [17–21]. Due to its flexibility in design, numerous PCF-based sensor structures, including elliptical [22], hexagonal [23], octagonal [24], rectangular [25] and others, have been described. In order to detect illegal drugs or other compounds, a number of researchers have recently developed different PCF shapes in liquid specimens. But in order to lower PCF structural loss [26,27], every study has used an excessive amount of background materials, including Teflon, Topas, and Zeonex [28,29]. THz radiation therapy has had extensive effect on PCF development devices because it offers previously unheard-of opportunities for extremely sensitive but secure observation. Because of the unique fiber form, THz wavelengths that employ PCF sensing perform well and offer accurate measurements and component analysis for a range of applications. Due to their ability to both see minute characteristics and penetrate imperceptible coverings, THz photons are quite beneficial for purposes such as drug testing, medical image processing, and security audits. Thanks to a diverse technique combined with the electronically controlled dispersion features of PCF, THz-based technologies may precisely quantify topic content, width, and RI [30–32]. Spectroscopy [33], sensors [34], astronomy [35], communication fields [36], and many other domains are said to have used for terahertz technology in recent years. Beams must have strong directional quality in order to reach high degree of performance [37]. Usually, the region of 0.1–10 THz is thought to contain terahertz waves, often known as T-rays [38]. Due to its location between the infrared and microwave spectrums, this radiation band is frequently used without causing harm to people or the environment [39]. It’s recommended above X-ray range because the electromagnetic range contains no dangerous radiation. Electromagnetic waves at high frequency, such as gamma and x-rays, have significant disadvantages even though they may travel across long distances. Gamma and X rays may easily ionise metals and biological tissues, that can result in a number of potentially fatal illnesses in both humans and other animals. As an alternative, electromagnetic waves both ionized and non-ionized fall within the oscillation range of the THz spectrum [40]. To attain a sensitivity of 77.08% for benzene, 77.18% for ethanol and 77.23% for water respectively, Kanmani et al. [41] used a slotted core PCF with Polytetrafluoroethylene as background material. Bulbul et al. [42] reports that at 1.6 THz frequency, a circular core illegal drugs sensor with a hexagonal cladding achieved a maximum sensitivity of 90.72%, 92.34%, 94.91%, 97.84% of cocaine, amphetamine, ketamine and morphine respectively. For liquid sensing purposes, Hossain et al. [43] suggested a PCF sensor using hexagonal core at 1 THz in 2022 in order to obtain RS of 88.70% and low CL 6.11 × 10−8 dB/m under optimal condition. An illegal drug sensing sensor was given by Monir et al. [44], who proposed that for three compounds ketamine, amphetamine, and cocaine the sensitivity at 1 THz was 85.50%, 89.50%, 90.20%. A PCF sensor with circular core and elliptical cladding was created by Pandey et al. [45] that operates in the THz regime and has sensitivities of 93.34%, 93.39%, and 97.22% for liquid samples of amphetamine, ketamine and cocaine within a functional wavelength of 1.8 µm.

In this research an HC-PCF based biosensor with precise, cutting-edge, environmentally friendly, and cost-effective characteristics has been developed and modeled to detect illicit drugs in blood. With a hybrid air hole in cladding of different sizes, an innovative simple circular core HC-PCF has been built. The model’s background material is Zeonex. Low EML and CL are displayed by the suggested sensor type, and the effective area has considerable values. Moreover, this model has increased sensitivity, which is advantageous for identifying cocaine, amphetamine, and ketamine, respectively. For drug detection applications, sensitivity is one of the most important guiding factors. With a sensitivity of 99.92%, 99.12% and 98.83%, this HC-PCF can reliably identify substances with low CL of 1.275 × 10^−7^ dB/m, 2.653 × 10^−9^ dB/m, and 4.106 × 10^−10^ dB/m, and low EML of 0.0042 cm^-1^, 0.0044 cm^-1^ and 0.0045 cm^-1^ for ketamine, amphetamine and cocaine respectively. Thus, biosensing and other sensing applications will benefit from this study of HC-PCF structure.

## 2. Methodology

The proposed HC-PCF sensor structure was selected based on a strategic balance between light-analyte interaction, fabrication feasibility, and optical performance. Specifically, we chose a simple circular hollow core surrounded by a three-layer cladding with hybrid circular and wind turbine-shaped air holes to achieve the goals Maximized Relative Sensitivity (RS), Minimized Optical Losses (EML & CL), Improved Fabrication Potential, and Design Optimization Process.

Using COMSOL Multiphysics V6.1 Simulating Programs, we optimized key parameters — core radius (pitch), air hole radii (Rx, Ry, Rz, Rt), and spacing (L1–L4) — based on performance metrics such as RS, CL, EML, EA, NA, and Spot Size. After evaluating multiple performance across design variants, we identified the optimal configuration that achieved high sensitivity (>99%) with low loss at a pitch of 100 µm and operating frequency of 1.6 THz.

**Fig 1:**
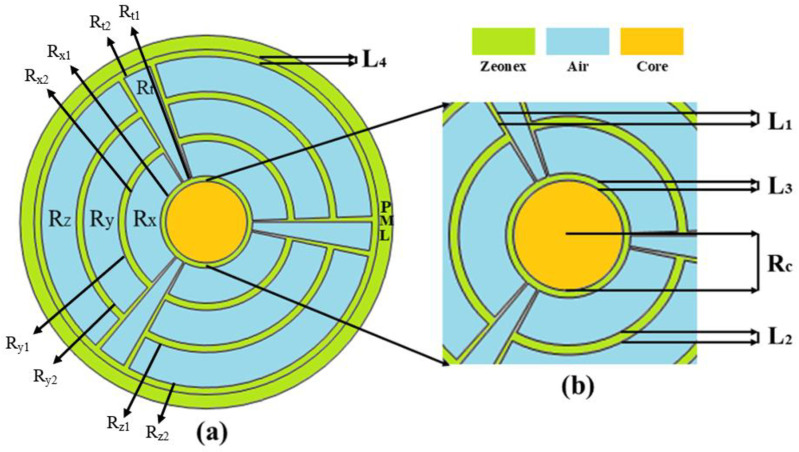
(a) A two-dimensional view of the proposed Illegal Drugs Sensor. (b) The suggested sensor’s circular core.

Hybrid air channels comprise the specially designed scanner’s coating region. At the center of this PCF is a circular area. These cores ease of deception is one of their advantages. Zeonex serves as the background substance. On the clad layer, a total of 12 composite air intakes are created. Ketamine, Amphetamine, and Cocaine are considered to be the indicated prohibited medicines in this instance. Here, the cross section of the suggested PCF is shown in Fig 1. Fig 1 has been updated to include dimension labels and material details to ensure consistency with the text and improve clarity for readers. Each parameter — such as the inner and outer radii of the cladding air holes (Rx1–2, Ry1–2, Rz1–2, Rt1–2), inter-layer spacing (L1–L4), and core radius (Rc) — is now clearly annotated. To enhance visual clarity, distinct colors have been used to represent different materials and structural regions: light green indicates the Zeonex background material, light blue denotes the air holes in the cladding layers, and light yellow is used to represent the hollow core region where analytes are introduced.

The radius of the core is expressed as R_C_ = 1.159P. In the first cladding layer, the inner radius of the air hole R_x1_ = 1.31P, whereas the outside radius is R_x2_ = 2.31P. The inner layer radius of the second cladding layer is R_y1_ = 2.51P, while the outer radius of the air holes is R_y2_ = 3.51P. There is a third layer of cladding on the structure. This layer has an air hole with inner radius of R_z1_ = 3.71P and outer radius of R_z2_ = 4.71P. Additionally, this PCF design features cladding in the style of a wind turbine. The exterior layer of this cladding is R_t2_ = 4.71P, while the inner layer is R_t1_ = 1.31P. Additionally, the two air holes angle distance is L_1_ = 2º, and intervening space between the two cladding is L_2_ = 0.2P. Furthermore, the distances L_3_ = 0.15P is found between the core and first cladding inner layer and L_4_ = 0.2P is found between the last cladding outer layer and PML. In addition, the inner and outer radius of PML is 4.19P and 5.32P and determined with P varying between 90 µm to 150 µm. PML covers the outer rim of the simulation and absorbs all incoming waves. Zeonex was selected as the background waveguide material due to its exceptional qualities that make it suitable for terahertz (THz) applications. These qualities include biocompatibility, high thermal stability, steady refractive index (1.53), and low absorption loss. These features guarantee strong light confinement, little effective material loss (EML), and dependable sensor performance for detecting illicit substances including cocaine, amphetamine, and ketamine. Furthermore, Zeonex is suitable for complicated PCF constructions due to its mechanical strength and compatibility with production methods including extrusion and 3D printing. The suggested HC-PCF sensor design depends on a low-loss, high-sensitivity environment, which is maintained by its use.

**Fig 2:**
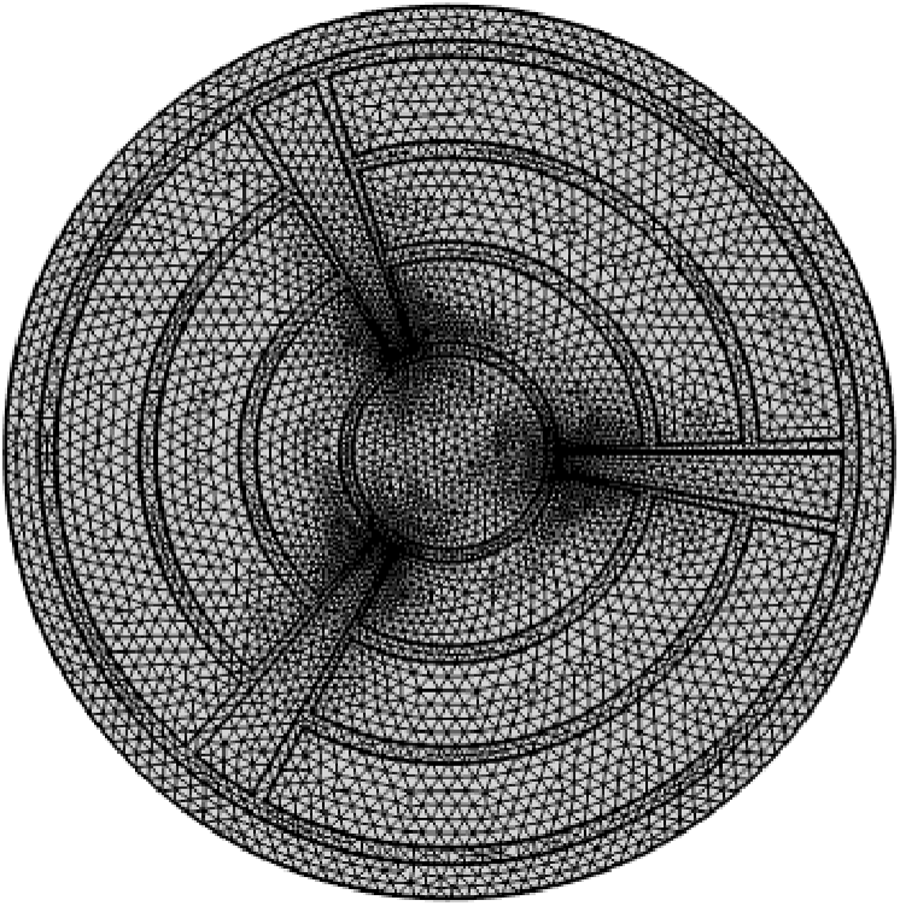
Mesh layout of proposed PCF.

Mesh structures create periodic air-hole patterns in the cladding, they are crucial to the building of a PCF. We are using finer mesh configuration by Comsol Multiphyscis V.6.1 software. To mess with the suggested design, physics-controlled messing and finer element size are selected. Fig 2 depicts the computational messing domain with 78 vertex elements, 1025 boundary elements, 5496 elements, and 0.3963 minimal element quality.

When the recommended sensor is loaded with the illegal drugs ketamine, amphetamine and cocaine by all the figures, there is a tight constraint on the light within the core region, which is necessary for better performance.

**Fig 3:**
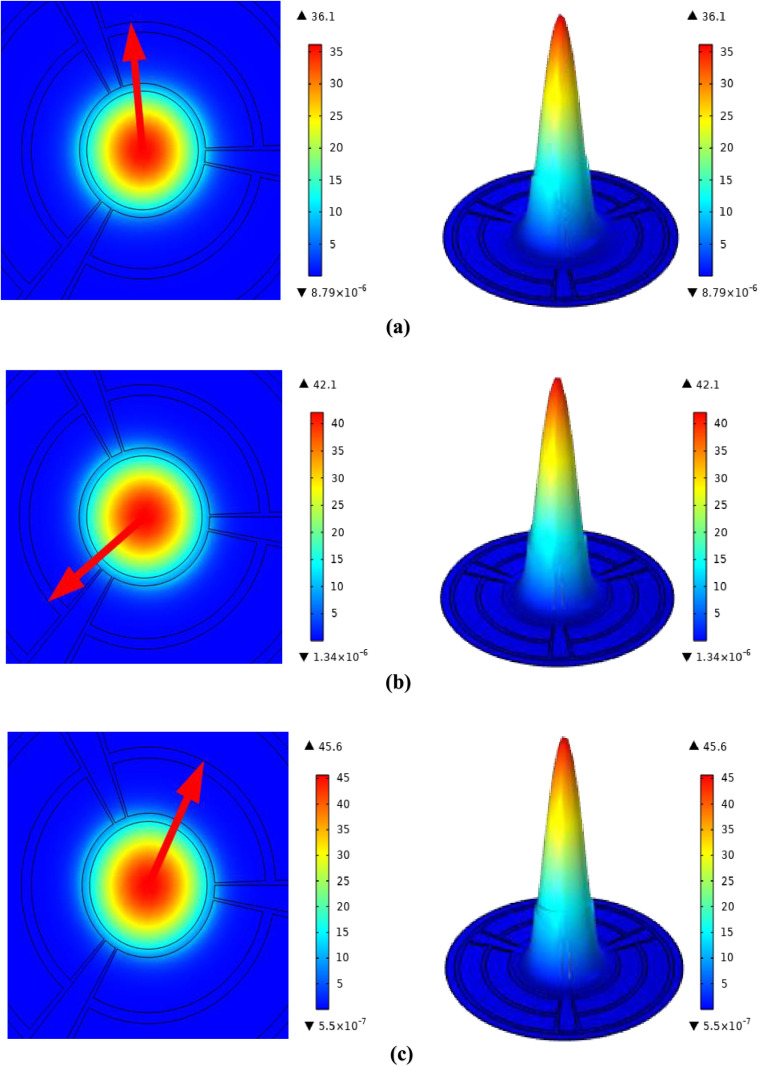
Electric field and density distribution for (a) Ketamine, (b) Amphetamine, (c) Cocaine.

The simulated electric field and energy density distributions in the core region of the suggested PCF sensor are shown in Fig 3 when it is loaded with the following illegal drug analytes: (a) cocaine, (b) amphetamine, and (c) ketamine. The operating frequency at which these field profiles were acquired was 1.6 THz. The electromagnetic field is tightly contained within the hollow core, as demonstrated, and there is a substantial interaction between the analyte medium and the directed light. Achieving high relative sensitivity requires this tight confinement since the sensor’s capacity to detect changes in refractive index is maximized by a larger overlap between the light field and the analyte. Additionally, the distributions show that the field maintains a steady and uniform propagation pattern, guaranteeing strong sensing capability, even in the face of minor fluctuations in analyte refractive indices.

Table 1 lists the refractive indices of the illegal drug samples which were used in the simulation to model their interaction with the guided THz wave within the PCF sensor.

**Table 1:** Several drug samples of refractive index [45].

Drug sample	Refractive index
Ketamine	1.562
Amphetamine	1.518
Cocaine	1.5022

## 3. Numerical and mathematical methods

The most crucial characteristics of a sensor are its relative sensitivity and its ability to indicate the concentration of analytes that contains the particular sensing elements. Usually, the refractive index is matched to confirm the sensing. To evaluate relative sensitivity, the light intensity that directly interacts with the detecting analyte must be computed [46]. It is possible to calculate the relative sensitivity (r) of analytes using equation (1) [47].


$$\begin{array}{c} r=\;\frac{n_{r}}{n_{eff}}\times k\% \end{array}$$ (1)

n_eff_ in this instance indicates the effective refractive mode index, whereas the analyte’s RI is indicated by n_r_. The power ratio is represented by k The amount of light connected to the sensor materials in the core is measured using the power ratio (k). It can be assessed using the subsequent equation (2) [47].


$$\begin{array}{c} \;k=\frac{\int sample\;R_{e}\left(E_{m}H_{n}-E_{n}H_{m}\right)dxdy}{\int total\;R_{e}\left(E_{m}H_{n}-E_{n}H_{m}\right)dxdy}\times 100\% \end{array}$$ (2)

The components of the magnetic and electric fields are represented, respectively, by E_m_, E_n_, and H_m_, H_n_. x and y represent the orientations of the polarisation modes.

Owing to fiber material’s characteristic, light waves are attenuated as they travel through PCF. An important task facing the scientists working in this sector is designing a fiber with little loss. EML is measured using the method below equation (3). It is sometimes referred to as material absorption loss or the major loss mechanism [48].


$$\begin{array}{c} \alpha_{eff}=\frac{({\frac{\varepsilon_{0}}{\mu_{0}})}^{\frac{1}{2}}\;\int A_{\max}\;n\alpha_{mat}{\left|E\right|}^{2}dA}{2\int ALL\;S_{z}dA}\; \end{array}$$ (3)

where n is the RI used for material, ε_0_ is the vacuum’s permittivity, μ_0_ is its permeability, α_mat_ is the bulk material absorption loss, S_z_ is the z-component of the Pointing vector, and E is the electric field’s amplitude.

Another significant kind of loss that mostly affects fibers made from raw materials is confinement loss. Single mode fibres are mostly impacted by confinement loss, also known as leakage loss, which is the loss of power between the core and the cladding. Because the core’s refractive index is equal to that of the outer cladding without an air hole, PCFs with guided modes are leeky. In the case of PCF, an unlimited number of air holes on the cladding should theoretically allow for lossless transmission. However, especially since the photonic crystal coating only contains a few air holes, leaky modes are present in practice-manufactured fibers. Confinement Loss can be measured by equation (4) [49,50]:


$$\begin{array}{c} L_{c}=8.686\left(\frac{2\pi f}{c}\right)Im\left(n_{eff}\right)\; \end{array}$$ (4)

where c is the light velocity in free space, L_c_ is the confinement loss, f is the operating frequency, and Im(neff) is the imaginary value of effective RI.

Equation (5) for the detection of illicit substances (cocaine, amphetamine, and ketamine) establishes the effective mode area of the proposed PCF [51]:


$$\begin{array}{c} A_{eff}=\;\frac{{[\int I\left(r\right)rdr]}^{2}}{{[\int I^{2}\left(r\right)rdr]}^{2}}\;\backslash \; \end{array}$$ (5)

in which the electric field intensity is represented by I(r) = |Et|^2^ and r is the core radius.

It is also necessary to take the suggested sensor’s NA properties into account. Although a lot of earlier research has not addressed it, the NA is a crucial characteristic for any type of optical sensor. The NA provides details on the incident light’s angular acceptance that the optical fiber is capable of passing through. The mathematical formula of Equation (6) is used to determine the NA [52]:


$$\begin{array}{c} \;NA=\frac{1}{\sqrt{1+\frac{{\pi A}_{eff}f^{2}}{c^{2}}}}\approx \frac{1}{\sqrt{1+\frac{{\pi A}_{eff}}{\lambda^{2}}}}\;\; \end{array}$$ (6)

where A_eff_ stands for the effective area and c is the speed of light in open space.

For any sensor, the spot size is a critical metric. The Marcuse formula, found in equation (7) [53], can be used to investigate the connenction among spot size and V. The following method may be used to evaluate the spot size W_eff_:


$$\begin{array}{c} W_{eff}=R\;\times (0.65\times 1.619\times V^{-1.5}+2.879\times V^{-6}\; \end{array}$$ (7)

Here, the core radius is shown by R, while the normalised frequency is represented by V.

## 4. Results and discussion

Analytes or target samples of various kinds are injected into the sensor’s core, one at a time and separately. Then, we obtain various values from the light propagation via the suggested HC-PCF core region. Even when analytes have similar RIs, our numerical results demonstrate that the RS, EML, CL, EA and spot size vary distinctly due to subtle shifts in optical field confinement and propagation characteristics. In future experimental phases, signal processing techniques or multi-parameter analysis (e.g., combining all responses) can be employed to further improve selectivity.

The interaction between the substance in the surrounding air holes and the light (or electromagnetic field) passing through the fiber is frequently what determines the sensitivity of the sensor. Greater interaction between the evanescent field which reaches beyond the core into the cladding or air holes and the surrounding environment is made possible by the porous structure created by these air holes. The parameters of the sensor (such as effective material loss, refractive index, or absorption) would change as a result of interactions between the analytes and the evanescent field that extends from the core into the surrounding air holes. The target analytes may then be accurately identified and quantified as this interaction would be detectable.

**Fig 4:**
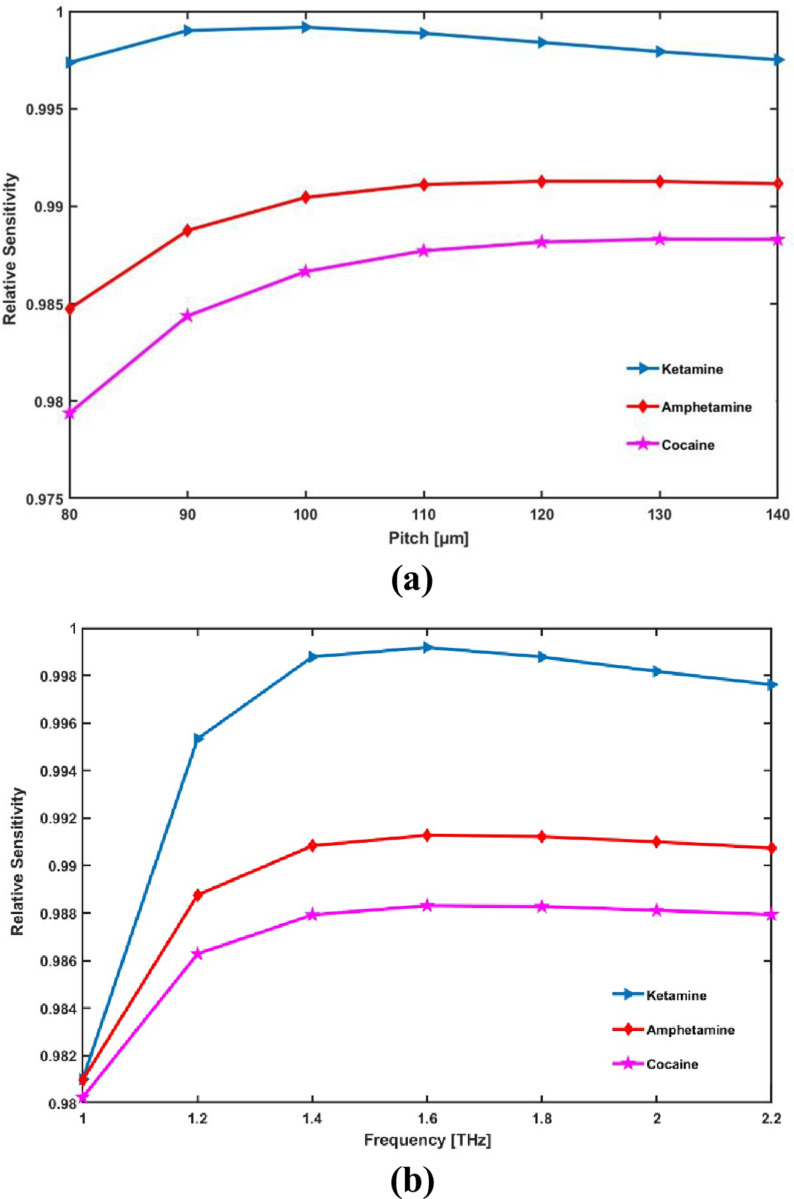
Display the effects of (a) pitch [ **μ**m] and (b) frequency [THz] on RS.

Fig 4(a) illustrates the RS graphically for each of the three elements under various geometric conditions at a constant frequency. The ensuing diagram illustrates that when the core radius increases from 80 μm to 140 μm, the relative sensitivity rises and as the core radius continues to climb the sensitivity decreases. Under ideal circumstances, the suggested sensor provides exceptionally high RS values of 99.92%, 99.12% and 98.83% for ketamine, amphetamine, and cocaine respectively. Furthermore, Fig 4(b) reports the variation in the suggested sensor’s relative sensitivity at various operating frequencies under ideal circumstances. According to the provided graphical representation, the relative sensitivity rises at a margin as the frequency increases, and then the sensitivity reaches saturation. As illustrated in the figure, the RS for all analytes reaches a maximum at 1.6 THz and thereafter becomes nearly constant.

The sensitivity initially increases with core radius because a larger core improves light-analyte interaction, but beyond a certain size, light spreads out, reducing sensitivity. As frequency increases, sensitivity rises due to better light confinement, but it eventually saturates at 1.6 THz when further increases no longer enhance interaction. Consequently, we will emphasize the results with a 100 μm core radius and 1.6 THz as the operational frequency in the ensuing discussion.

**Fig 5:**
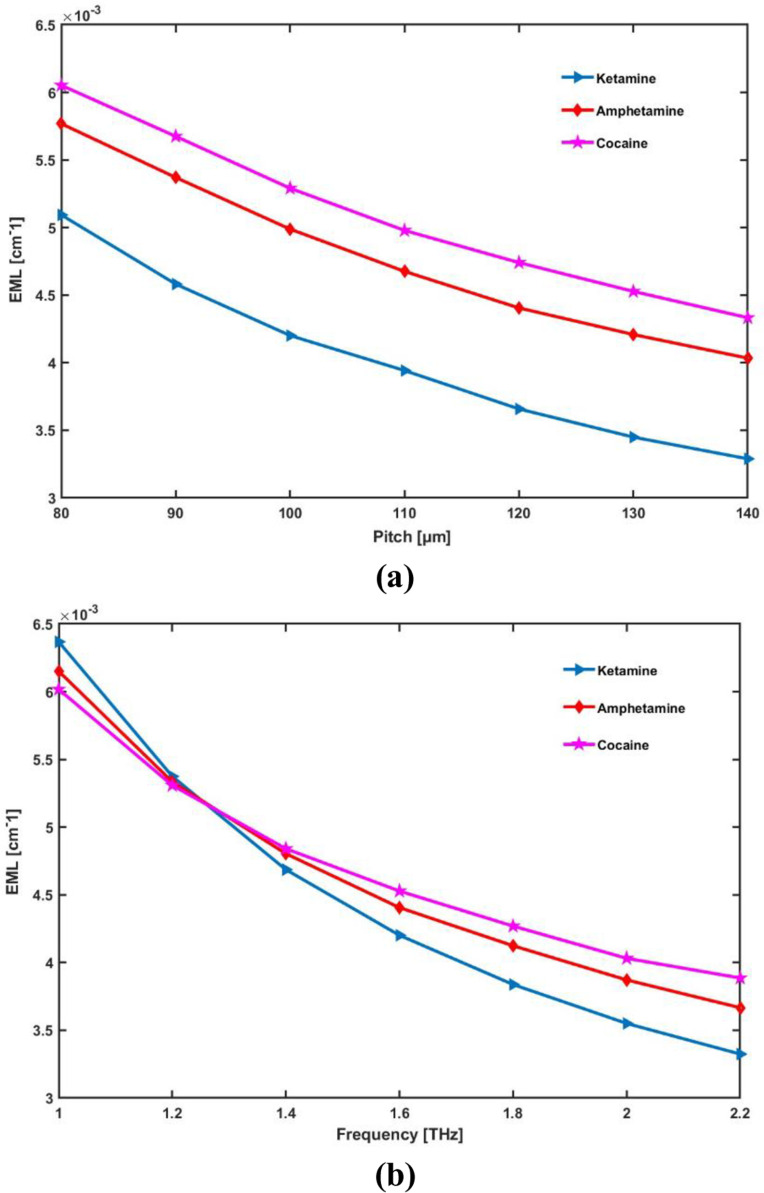
Display the effects of (a) pitch [ **μ**m] and (b) frequency [THz] on EML.

Plot relations for EML with various core sizes and frequency are shown in Fig 5:

As core radius (or pitch) increases, EML decreases because more light is confined within the core, reducing material absorption. However, beyond an optimal size, light can leak, causing EML to rise. Similarly, increasing frequency reduces EML by confining light more effectively and minimizing material interaction, but at very high frequencies, EML may stabilize or increase due to dispersion effects.

Fig 5(a) shows that an increase in core radius results in a decrease in effective material loss. Furthermore, the flexibility of the THz application and its fiber thickness are both significant factors. For compactness and flexibility, the sensor should have the thinnest possible diameter [53]. Thus, the ideal core radius that we have selected is 100 μm. Fig 5(b) displays the sensor’s EML once more for various operating frequencies at 100 μm core radius. The EML is very low over a broad frequency range of 1.0–2.2 THz, as can be shown in Fig 5(b). Thus, any kind of long-distance communication might use this fiber. Under perfect circumstances, the suggested hollow core PCF-based sensor provides remarkably low effective material loss values for ketamine, amphetamine and cocaine respectively of 0.0042 cm-1, 0.0044 cm-1 and 0.0045 cm-1.

**Fig 6:**
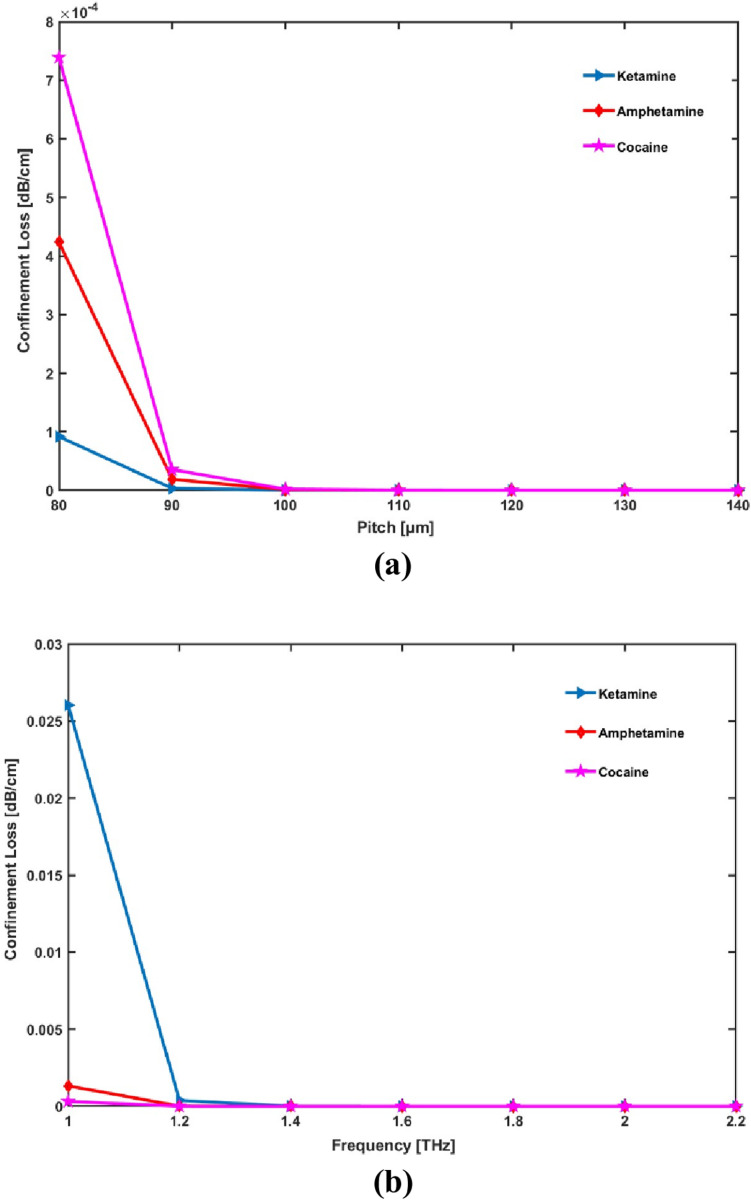
Display the effects of (a) pitch [ **μ**m] and (b) frequency [THz] on CL.

Plot relations for CL with various core sizes and frequency are shown in Fig 6:

Confinement loss (CL) initially decreases as the core radius (or pitch) increases due to improved light confinement, reducing interaction with the fiber material. However, beyond an optimal size, CL may stabilize or increase due to light leakage. Similarly, at lower frequencies, CL is higher because of greater material interaction, but as frequency increases, CL decreases due to better confinement, eventually stabilizing as further frequency increases yield minimal improvements.

At first, when the suggested sensor’s operating frequencies and core radius increase, the confinement loss decreases quickly. After that, as shown by both curves, Fig 6a and 6b respectively, it fattens out and practically remains unchanged despite an increase in the operating frequencies and core size. At ideal conditions, the proposed hollow core PCF-based sensor provides exceptionally low CL of 2.75 × 10^−7^ dB/m, 2.653 × 10^−9^ dB/m, and 4.106 × 10^−10^ dB/m for ketamine, amphetamine, and cocaine respectively.

**Fig 7:**
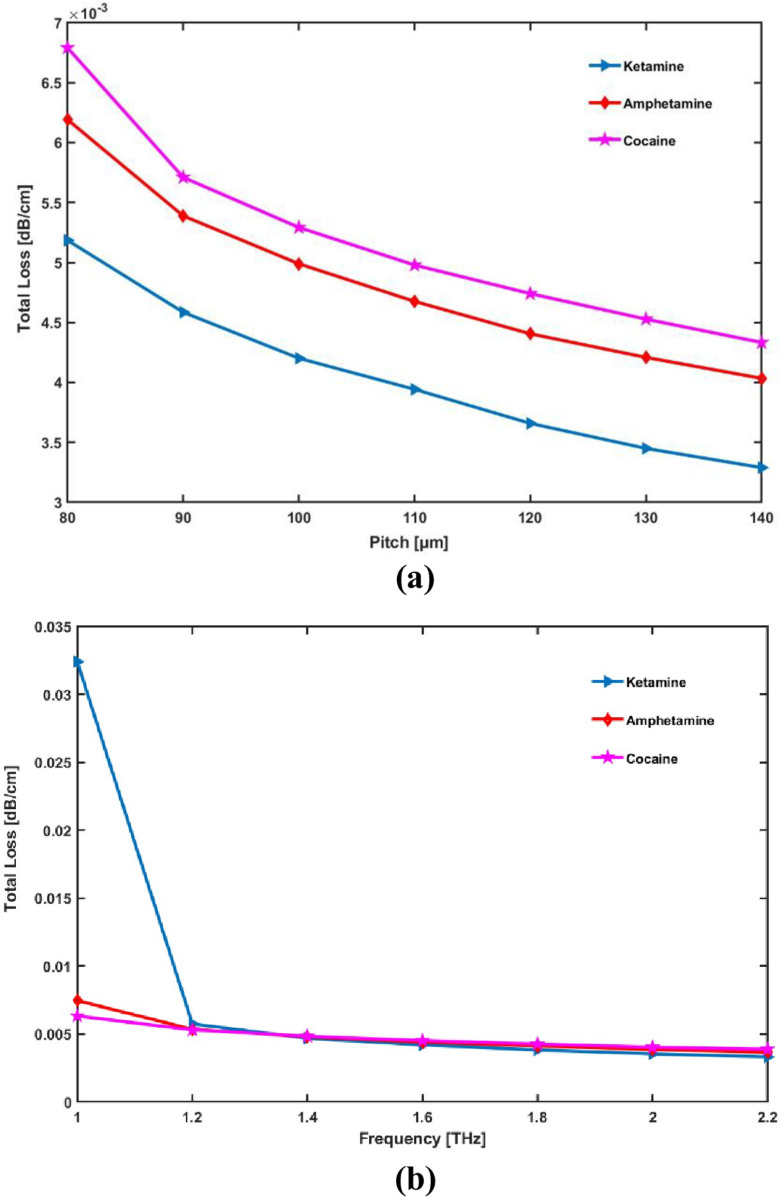
Display the effects of (a) pitch [ **μ**m] and (b) frequency [THz] on Total Loss.

As shown in Fig 7a–7b for different geometrical parameters and operating frequencies, the total loss for the proposed drug sensor is the consequence of combining EML and CL.

Fig 7a indicates a notable decrease in the overall loss as a consequence of the initial increase in core radius. Additionally, as Fig 7b illustrates, higher frequencies reduce total loss.

As pitch (or core radius) grows, total loss reduces because a bigger core contains more light, which lowers absorption and material interaction. Similarly, by strengthening light confinement inside the core, higher frequency lowers overall loss. Less total loss results from more effective light direction caused by both higher frequency and greater pitch.

The effective area increases with pitch (or core radius) because a larger pitch allows light to spread more within the core, enhancing energy density for nonlinear effects. Conversely, as frequency increases, the effective area decreases due to improved light confinement, resulting in a smaller mode area. This relationship highlights how pitch enhances interaction with analytes, while frequency optimizes light guidance within the fiber.

**Fig 8:**
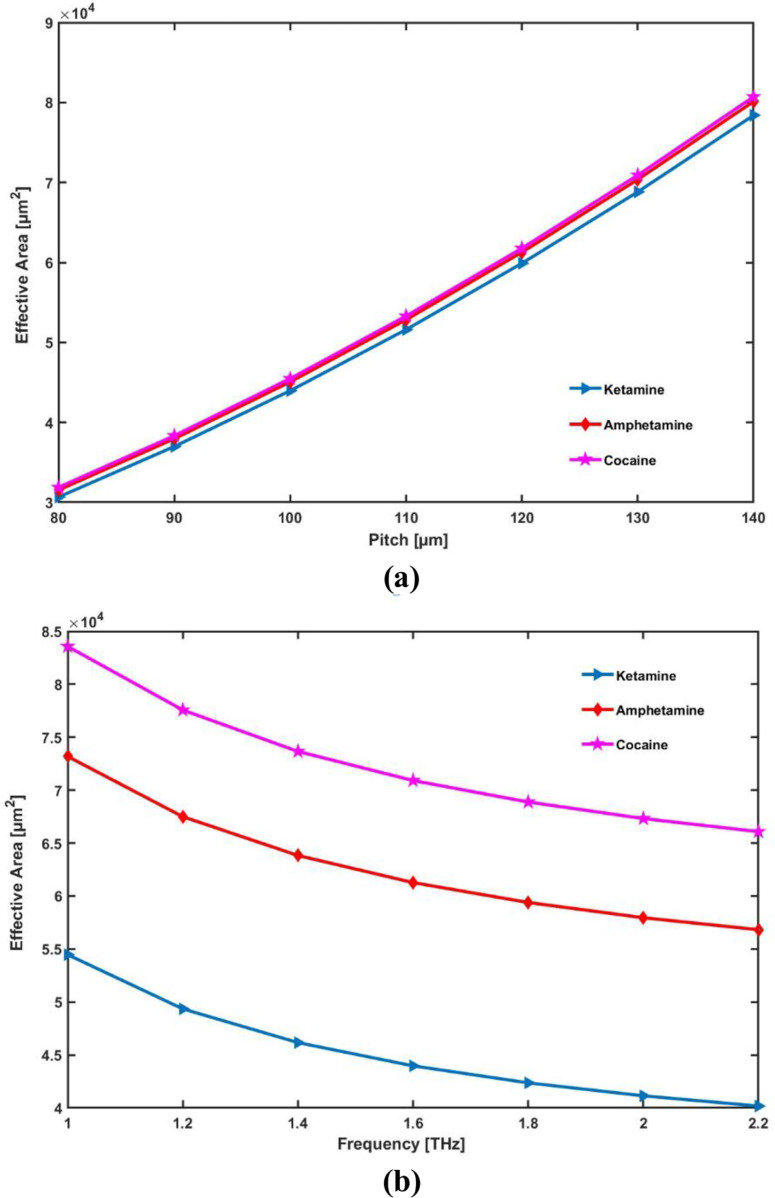
Display the effects of (a) pitch [ **μ**m] and (b) frequency [THz] on EA.

At Fig 8(a) and 8(b) it shows the effective area which increases continually as pitch is increased and decreases as frequency is increased. This permits a very high density of energy of optical light transmitted along the innermost region of the PCF required for non-linear effects and consequently a large relative sensitivity of confined. This results in a smaller efficient mode area due to the fact that the optical light is confined within the core region. Ketamine, amphetamine, and cocaine respectively effective areas of 4.40 × 10^4^ μm^2^, 6.13 × 10^4^ μm^2^ and 7.09 × 10^4^ μm^2^ determined at 1.6 THz.

The fiber needs to be able to accept the maximum quantity of light from the source, hence the NA is a unit less parameter must have the highest achievable value.

Numerical aperture (NA) decreases as pitch (or core radius) increases because a larger core spreads the light, reducing its confinement and narrowing the acceptance angle. Similarly, as frequency increases, NA decreases due to stronger light confinement within the core, which also reduces the acceptance angle. Thus, both larger pitch and higher frequency result in lower NA.

**Fig 9:**
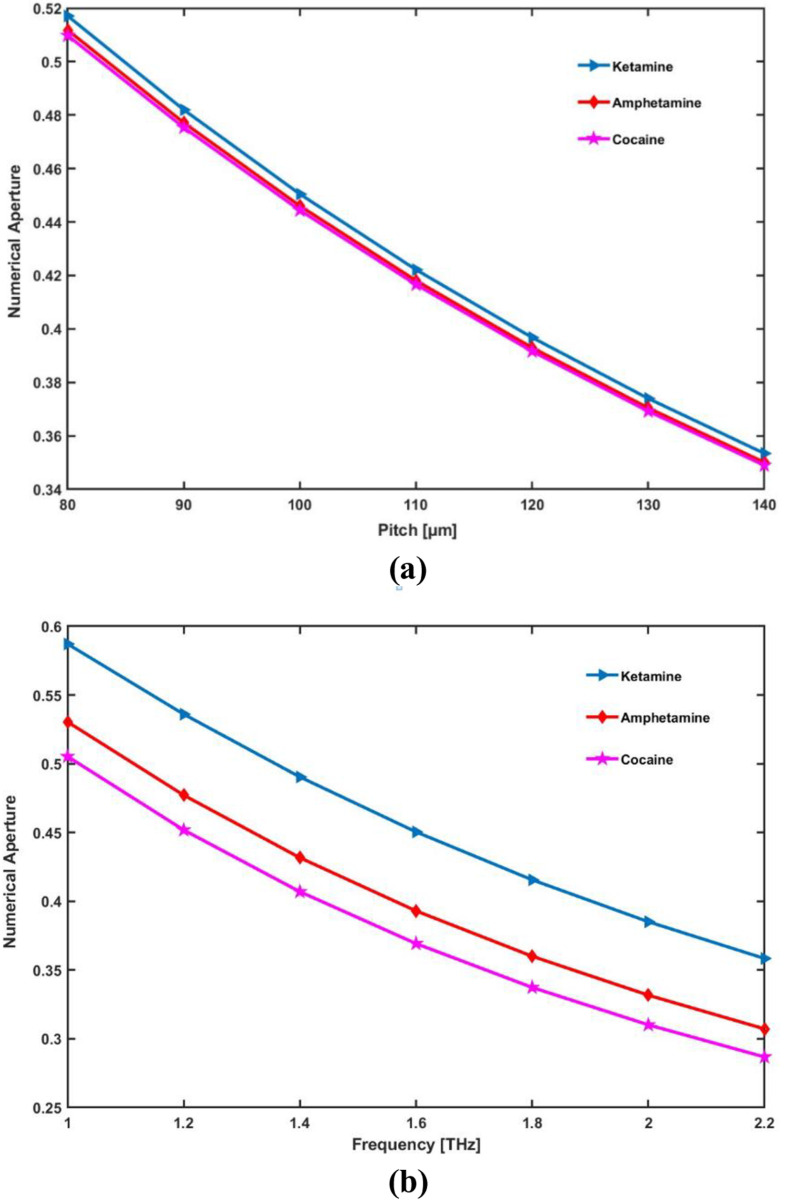
Display the effects of (a) pitch [ **μ**m] and (b) frequency [THz] on NA.

Fig 9 reports the relationship between numerical aperture with pitch and frequency. NA and pitch have an inverse relationship, the NA decrease as pitch increases. Also, NA decrease as frequency increases. When the geometry is ideal, the fiber that is being provided offers a favorable NA of 0.450, 0.393, 0.369 for ketamine, amphetamine, and cocaine respectively at 1.6 THz.

The spot size increases as pitch (or core radius) increases because a larger core allows light to spread over a wider area. In contrast, the spot size decreases with increasing frequency, as higher frequencies better confine the light within the core, concentrating it into a smaller area. Thus, pitch enlarges the spot size, while frequency reduces it.

**Fig 10:**
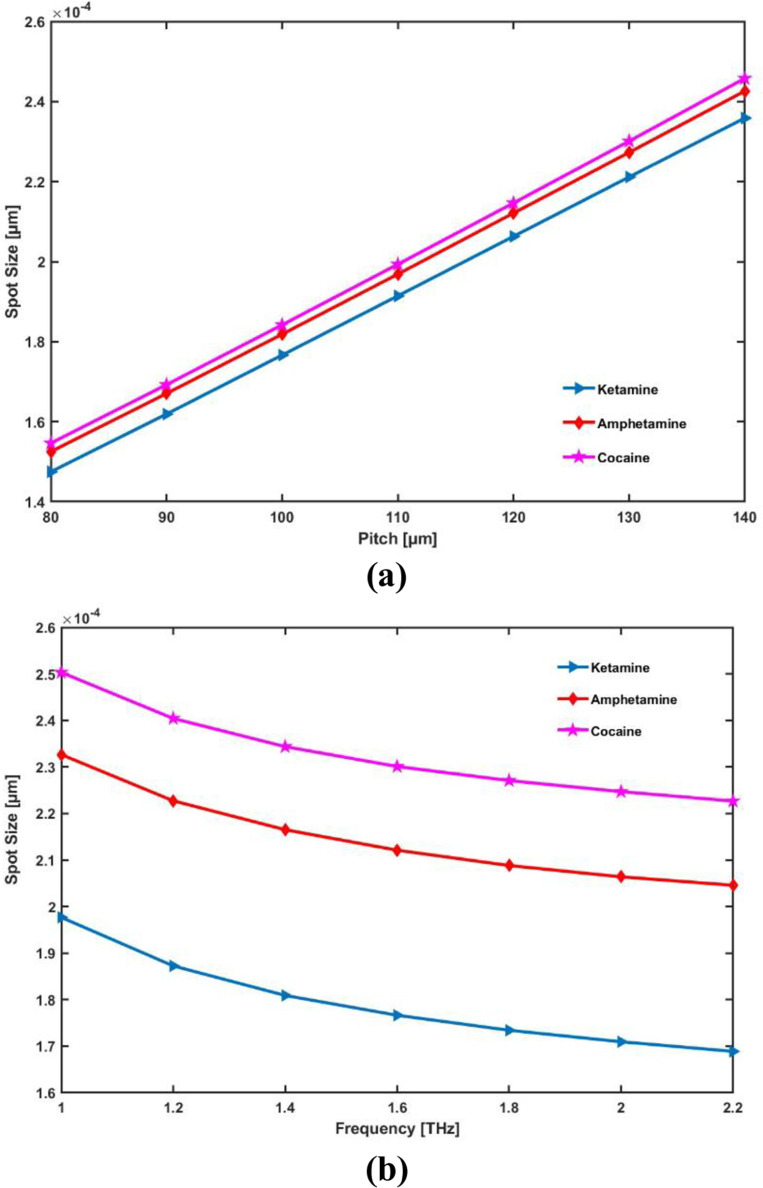
Display the effects of (a) pitch [ **μ**m] and (b) frequency [THz] on Spot Size.

As the core radius varies, Fig 10(a) shows how the spot size of the suggested detector varies. As PCF pitch rises, the spot size rises proportionately. Variations in the PCF sensor’s operating frequency cause changes in the spot size, as shown in Fig 10(b). The working frequency increases from 1.0 to 2.2 THz, causing the spot size to shrink. In ideal conditions, the spot size of the suggested sensor is 1.77 × 10^−4^ µm for cocaine, 2.12 × 10^−4^ µm for amphetamine, and 2.30 × 10^−4^ µm for ketamine.

**Fig 11:**
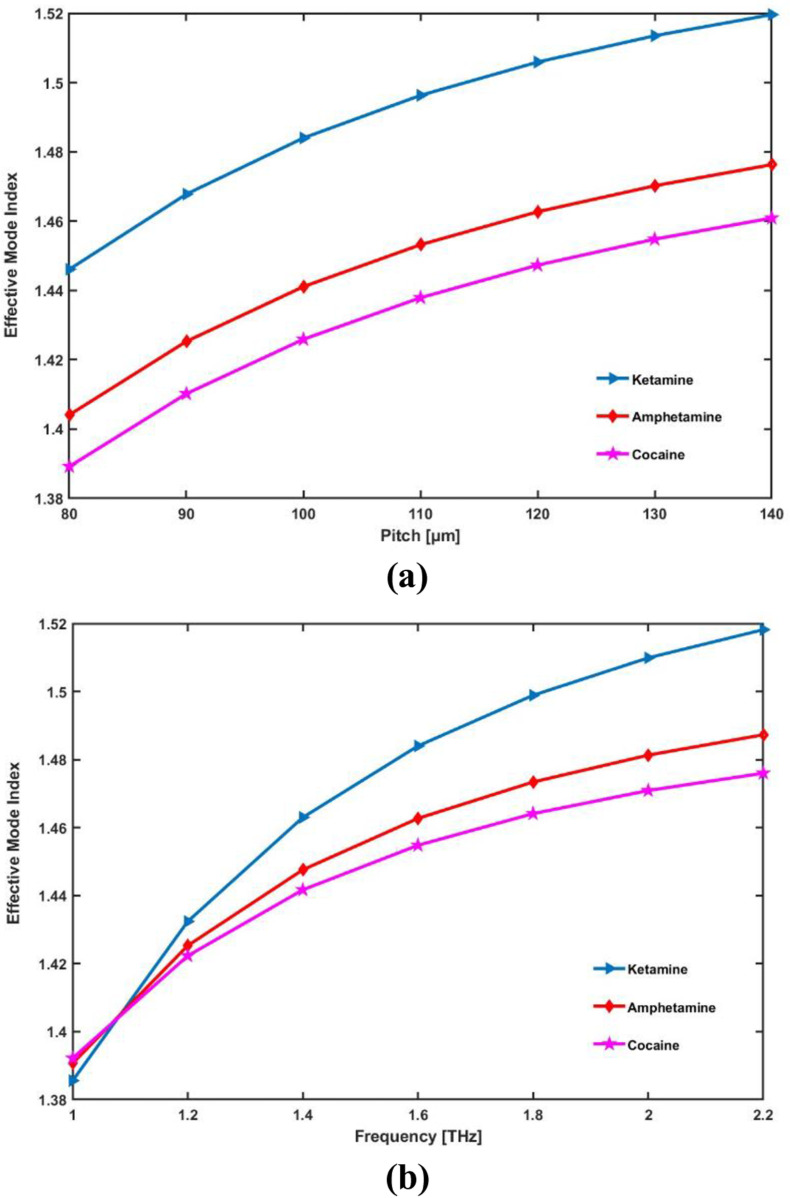
Display the effects of (a) pitch [ **μ**m] and (b) frequency [THz] on Effective Mode Index.

To further illustrate the optical behavior of the proposed PCF sensor, we have included a dispersion relation plot in Fig 11 showing the variation of the effective mode index (n_eff_) with respect to pitch and frequency in the terahertz range. This plot demonstrates how the modal refractive index evolves as the frequency changes from 1.0 THz to 2.4 THz, offering insights into the dispersion characteristics of the guided mode. As observed, the n_eff_ exhibits a smooth and monotonic trend with minimal fluctuation, indicating stable modal propagation across the operating frequency band. The relatively flat dispersion curve near the optimal operating frequency of 1.6 THz confirms the sensor’s low-dispersion regime, which is ideal for maintaining signal integrity and enhancing sensitivity. When the geometry is ideal, the fiber that is being provided offers a favorable n_eff_ of 1.484, 1.463, 1.455 for ketamine, amphetamine, and cocaine respectively at 1.6 THz.

The sensor design parameters, such as the geometry, core structure, and material choices, have been carefully selected to match practical fabrication capabilities like stack-and-draw, extrusion, and 3D printing methods, which have been successfully used in previous PCF fabrications. Additionally, the physical characteristics such as extremely low confinement loss, high relative sensitivity, and manageable effective material loss fall within ranges that are achievable with current terahertz measurement technologies. The integration of a perfectly matched layer (PML) boundary in simulations mimics real-world absorption conditions, providing a highly accurate prediction of device performance. Prior experimental studies on similar hollow-core PCFs with simpler or more complex structures have shown good agreement between simulation and fabrication results, further reinforcing confidence. Therefore, while experimental validation is a future step, the well-grounded numerical results and alignment with current fabrication technologies give strong assurance that the proposed sensor will work effectively under real experimental conditions.

**Fig 12:**
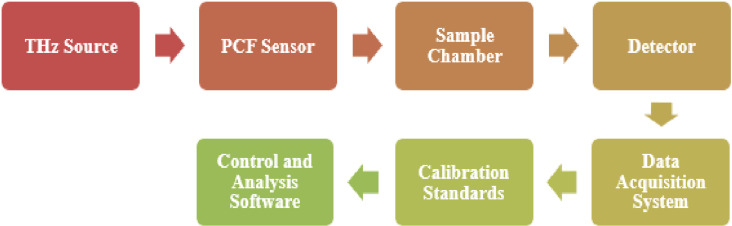
Display the proposed PCF sensor’s experimental flow chart.

Fig 12 illustrates the schematic of the experimental setup for the proposed terahertz (THz) photonic crystal fiber (PCF) sensor system. The system begins with a THz source, which generates the broadband THz signal that propagates through the PCF sensor. The sensor is connected to a sample chamber, where analytes (e.g., ketamine, amphetamine, cocaine) can be introduced and interact with the guided THz wave. The modulated signal is then transmitted to a THz detector, which converts it into measurable electrical data. This signal is processed by a data acquisition system, followed by comparison with calibration standards to ensure accurate identification. Finally, the control and analysis software manage system operation and performs spectral analysis for analyte detection.

This setup demonstrates a practical and integrated approach for real-time detection using the proposed PCF sensor. It is compatible with existing THz time-domain spectroscopy (THz-TDS) systems, and its modular design allows for straightforward alignment, calibration, and data interpretation. The sensor’s design is tailored to fit seamlessly into this system, making it suitable for experimental validation and future commercial applications.

## 5. Comparison with previous works

Illegal drugs sensing, including that of ketamine, amphetamine, cocaine, and other compounds, is important and in great demand in the industrial and real-world sectors. There aren’t many established techniques for determining drug concentration levels. On the other hand, they are expensive, time-consuming, and susceptible to mistakes. In this research to focus on PCF-based sensors in order to gauge the concentrations of drugs. A few authors have recently proposed several PCF-based sensors for illegal drugs identification, such as ketamine, amphetamine and cocaine. Based on a review of the literature and a tweak to the ideal settings for the proposed circular PCF sensor, Table 2 displays the sensing performance of these studies.

**Table 2:** Several comparisons between previously designed sensors and the proposed sensor.

Ref.	Year	Freq. (THz)	Elements	RS (%)	EML (cm^ − 1^)	CL (dB/m)	NA
[41]	2019	*f* = 1	Benzene	77.08%	0.0113	2.09 × 10^-12^	–
			Ethanol	77.18%	0.0122	9.07 × 10^-12^	–
			Water	77.23%	0.0141	2.47 × 10^−10^	–
[42]	2020	*f* = 1.6	Cocaine	90.72%	0.0096	2.558 × 10^-15^	–
			Amphetamine	92.34%	0.0117	1.402 × 10^-15^	–
			Ketamine	94.91%	0.0149	2.884 × 10^-16^	–
			Morphine	97.84%	0.0184	0	
[43]	2021	*f* = 1	Propanol	84.71%	–	5.70 × 10^−8^	–
			Ethanol	86.60%	–	5.75 × 10^−8^	–
			Butanol	88.70%	–	6.11 × 10^−8^	–
[44]	2023	*f* = 1	Ketamine	85.50%	–	6.40 × 10^−8^	
			Amphetamine	89.50%	0.02	6.20 × 10^−8^	
			Cocaine	90.20%	–	7.10 × 10^−8^	
[45]	2024	–	Cocaine	93.34%	–	1.154 × 10^-17^	–
			Amphetamine	93.39%	–	− 1.82 × 10^-16^	–
			Ketamine	97.22%	–	3.185 × 10^-13^	–
This PCF	2024	*f* = 1.6	Ketamine	99.92%	0.0042	1.275 × 10^−7^	0.450
			Amphetamine	99.12%	0.0044	2.653 × 10^−9^	0.393
			Cocaine	98.83%	0.0045	4.106 × 10^−10^	0.369

Earlier designs for detection of illicit drugs often exhibit lower sensitivity and limiting their detection accuracy and reliability. Moreover, many prior works focus primarily on sensitivity without fully optimizing other critical parameters such as effective mode index (EMI), numerical aperture (NA), effective area (EA), and spot size, which are essential for stable modal propagation and efficient light coupling. Some previously proposed geometries are also structurally complex or rely on materials that are less compatible with terahertz (THz) operation, making experimental realization more challenging.

The proposed design can be considered structurally fragile to some extent because the air regions occupy a large portion of the cladding compared to the solid Zeonex and the core, which can reduce the overall mechanical strength of the fiber. A high air-filling fraction makes the structure lightweight but also more delicate and prone to collapse during fabrication, especially under thermal or mechanical stress. Potential fabrication challenges include maintaining the uniformity and precise alignment of the complex hybrid air-hole structures, avoiding collapse or deformation of thin struts separating the air holes, and ensuring proper core formation during the drawing or extrusion processes. Techniques like stack-and-draw, sol-gel methods, or advanced 3D printing with precise temperature and pressure control are essential to successfully fabricate such intricate structures without damaging their optical performance. In contrast, this proposed work introduces multiple key innovations that set it apart, including a new hybrid PCF structure, superior performance metrics, comprehensive optimization strategy, fabrication-oriented design, and drug-specific field analysis and structural tuning. The novelty of this research lies in the strategic integration of a hybrid hollow-core PCF design featuring circular and wind turbine-shaped cladding air holes, optimized for ultra-sensitive and low-loss terahertz detection of illicit drugs. Unlike prior studies, this work achieves near-unity relative sensitivity through analyte-specific structural tuning, supported by a multi-parameter optimization framework. Furthermore, the proposed sensor balances cutting-edge performance with fabrication feasibility, positioning it as a robust and scalable solution for next-generation biochemical sensing applications.

## 6. Fabrication feasibilities

The PCF sensor may be made using a number of different manufacturing methods. Stack-and-draw, sol-gel, extrusion, drilling, capillary stacking, and 3D printing are the primary techniques to be used in these manufacturing processes [54–56].

The circular fiber constructions might be made using stack-and-draw, sol-gel, or drilling procedures. However, using the extrusion or 3D printing processes, both circular and non-circular structures may be produced. The Max Plank Institute’s H. Ebendorff-Heidepriem et al. provided the original manufacturing procedure [55]. Furthermore, a number of PCFs with various lattice geometries have been built. Slotted structured air holes have also been created by Atakaramians et al. [30] with the use of the extrusion method. Furthermore, asymmetrically formed PCFs’ air holes may be created using 3D printing technology. Examples of our suggested core with non-circular air holes may be printed using 3D printing or extrusion.

### Conclusion

An innovative PCF sensor for identifying illicit drugs is the suggested biosensor. With a small architecture, it can be used for sensing in optical waveguides with a range of 80 µm to 140 µm. The HC-PCF was developed with COMSOL Multiphysics software, integrating both the Perfectly Matched Layer (PML) and Finite Element Method (FEM). This work proposes a novel circular core PCF for terahertz sensing applications. The numerical studies at 1.6 THz verify that, this suggested illegal substances sensor offers very high sensitivity of 99.92%, 99.12% and 98.83% at ketamine, amphetamine, and cocaine respectively. Furthermore, the CL of the proposed HC-PCF is 2.75 × 10^−7^ dB/m, 2.653 × 10^−9^ dB/m, and 4.106 × 10^−10^ dB/m; the EML is 0.0042 cm^-1^, 0.0044 cm^-1^ and 0.0045 cm^-1^ and the numerical aperture is 0.450, 0.393, 0.369. The EA is 4.40 × 10^−8^ m^2^, 6.13 × 10^−8^ m^2^, and 7.09 × 10^−8^ m^2^ for illicit drugs, such as amphetamine, cocaine, and ketamine, respectively. With current technology, the suggested sensor is incredibly easy to implement and construct. Thus, we anticipate that this sensor will be a formidable competitor for applications involving the detection of illicit narcotics. Numerous additional fields, including chemical sensing, biomedical, industrial, and food evaluation classes, will find it helpful.

## Supporting information

S1 File Frequency. (PDF)

S2 File Pitch. (PDF)
