# Supplementary material for: Illegal drugs sensor: Performance evaluation and identification based on terahertz photonic crystal fiber
Source: PLoS One. 2025 Jun 27;20(6):e0327013. doi: 10.1371/journal.pone.0327013 (PMC12204572; doi:10.1371/journal.pone.0327013)
Supplement: S1 File — (PDF) [file pone.0327013.s001.pdf]

wave	freq	p	nr	l	aff	real-x pol	real-y p
#DIV/0!							
3.00E-04	1.00E+12	1.00E-04	1.562	0.000153188	0.95	1.38560000000000	
2.50E-04	1.20E+12	1.00E-04	1.562	0.000153188	0.95	1.43240000000000	
2.14E-04	1.40E+12	1.00E-04	1.562	0.000153188	0.95	1.46300000000000	
1.88E-04	1.60E+12	1.00E-04	1.562	0.000153188	0.95	1.48400000000000	
1.67E-04	1.80E+12	1.00E-04	1.562	0.000153188	0.95	1.49890000000000	
1.50E-04	2.00E+12	1.00E-04	1.562	0.000153188	0.95	1.50990000000000	
1.36E-04	2.20E+12	1.00E-04	1.562	0.000153188	0.95	1.51820000000000	
[redacted]							
#DIV/0!							
#DIV/0!							
#DIV/0!							
3.00E-04	1.00E+12	1.20E-04	1.518	0.000183825	0.95	1.39070000000000	
2.50E-04	1.20E+12	1.20E-04	1.518	0.000183825	0.95	1.42530000000000	
2.14E-04	1.40E+12	1.20E-04	1.518	0.000183825	0.95	1.44760000000000	
1.88E-04	1.60E+12	1.20E-04	1.518	0.000183825	0.95	1.46270000000000	
1.67E-04	1.80E+12	1.20E-04	1.518	0.000183825	0.95	1.47340000000000	
1.50E-04	2.00E+12	1.20E-04	1.518	0.000183825	0.95	1.48130000000000	
1.36E-04	2.20E+12	1.20E-04	1.518	0.000183825	0.95	1.48730000000000	
1.25E-04	2.40E+12	1.20E-04	1.518	0.000183825	0.95	1.49190000000000	
1.15E-04	2.60E+12	1.20E-04	1.518	0.000183825	0.95	1.49560000000000	
1.07E-04	2.80E+12	1.20E-04	1.518	0.000183825	0.95	1.49850000000000	
#DIV/0!							
#DIV/0!							
3.00E-04	1.00E+12	1.30E-04	1.5022	0.000199144	0.95	1.39220000000000	
2.50E-04	1.20E+12	1.30E-04	1.5022	0.000199144	0.95	1.42230000000000	
2.14E-04	1.40E+12	1.30E-04	1.5022	0.000199144	0.95	1.44170000000000	
1.88E-04	1.60E+12	1.30E-04	1.5022	0.000199144	0.95	1.45480000000000	
1.67E-04	1.80E+12	1.30E-04	1.5022	0.000199144	0.95	1.46410000000000	
1.50E-04	2.00E+12	1.30E-04	1.5022	0.000199144	0.95	1.47090000000000	
1.36E-04	2.20E+12	1.30E-04	1.5022	0.000199144	0.95	1.47600000000000	
1.25E-04	2.40E+12	1.30E-04	1.5022	0.000199144	0.95	1.48000000000000	
1.15E-04	2.60E+12	1.30E-04	1.5022	0.000199144	0.95	1.48320000000000	
1.07E-04	2.80E+12	1.30E-04	1.5022	0.000199144	0.95	1.48570000000000	
wave	freq	p	nr	l	aff	real-x pol	

imag-x pol	imag-y p	area-x pol	area-y p	power-x pol	power-y p
					#######
1.4311000000000E-07		5.4465000000000E-08		8.7023000000000E-01	#######
1.7361000000000E-09		4.9347000000000E-08		9.1275000000000E-01	#######
2.5575000000000E-11		4.6163000000000E-08		9.3548000000000E-01	#######
4.3811000000000E-13		4.3968000000000E-08		9.4929000000000E-01	#######
8.4304000000000E-15		4.2362000000000E-08		9.5843000000000E-01	#######
1.6315000000000E-16		4.1137000000000E-08		9.6488000000000E-01	#######
8.7075000000000E-18		4.0172000000000E-08		9.6964000000000E-01	#######
[redacted]					
					#######
					#######
					#######
7.2170000000000E-09		7.3182000000000E-08		8.9872000000000E-01	#######
6.4254000000000E-11		6.7495000000000E-08		9.2838000000000E-01	#######
7.0633000000000E-13		6.3831000000000E-08		9.4488000000000E-01	#######
9.1402000000000E-15		6.1272000000000E-08		9.5517000000000E-01	#######
1.4760000000000E-16		5.9391000000000E-08		9.6210000000000E-01	#######
6.2637000000000E-18		5.7953000000000E-08		9.6704000000000E-01	#######
4.5031000000000E-18		5.6821000000000E-08		9.7070000000000E-01	#######
9.8611000000000E-18		5.5905000000000E-08		9.7351000000000E-01	#######
1.3274000000000E-17		5.5152000000000E-08		9.7570000000000E-01	#######
6.6919000000000E-18		5.4520000000000E-08		9.7746000000000E-01	#######
					#######
					#######
1.7263000000000E-09		8.3569000000000E-08		9.0847000000000E-01	#######
1.3206000000000E-11		7.7566000000000E-08		9.3383000000000E-01	#######
1.2584000000000E-13		7.3655000000000E-08		9.4814000000000E-01	#######
1.4140000000000E-15		7.0909000000000E-08		9.5713000000000E-01	#######
5.8945000000000E-18		6.8879000000000E-08		9.6321000000000E-01	#######
1.2215000000000E-16		6.7318000000000E-08		9.6753000000000E-01	#######
1.3174000000000E-17		6.6079000000000E-08		9.7071000000000E-01	#######
1.0574000000000E-17		6.5065000000000E-08		9.7312000000000E-01	#######
2.3083000000000E-17		6.4217000000000E-08		9.7499000000000E-01	#######
1.1992000000000E-18		6.3487000000000E-08		9.7645000000000E-01	#######
imag x pole		area x pole		power-x pol	

sens-x pol	sens y-p	EML-x pol	EML-y p	confinement -x pol
#DIV/0!	######		#######	#DIV/0!
0.9810185190531	######	0.0063678000000	#######	2.603418087887E-02
0.9953333566043	######	0.0053742000000	#######	3.789918923106E-04
0.9987831578947	######	0.0046857000000	#######	6.513547944714E-06
0.9991852964960	######	0.0042010000000	#######	1.275196420638E-07
0.9987775435319	######	0.0038377000000	#######	2.760543674560E-09
0.9981737598516	######	0.0035503000000	#######	5.935960604273E-11
0.9976140692926	######	0.0033239000000	#######	3.484898232172E-12
[redacted]				
#DIV/0!	######		#######	#DIV/0!
#DIV/0!	######		#######	#DIV/0!
#DIV/0!	######		#######	#DIV/0!
0.9809858057094	#REF!	0.0061504000000	#######	1.312896956207E-03
0.9887608503473	#REF!	0.0053339000000	#######	1.402669491880E-05
0.9908316109422	#REF!	0.0048037000000	#######	1.798910779976E-07
0.9912819170028	#REF!	0.0044053000000	#######	2.660416407732E-09
0.9912228858423	#REF!	0.0041238000000	#######	4.833178097896E-11
0.9909989333693	######	0.0038714000000	#######	2.278950440514E-12
0.9907366368587	######	0.0036661000000	#######	1.802221674337E-12
0.9905410416248	######	0.0035000000000	#######	4.305370599924E-12
0.9903133190693	######	0.0033594000000	#######	6.278401678177E-12
0.9901796996997	#REF!	0.0014804000000	#######	3.408642149852E-12
#DIV/0!	######		#######	#DIV/0!
#DIV/0!	######		#######	#DIV/0!
0.9802497011924	######	0.0060173000000	#######	3.140437876542E-04
0.9862894087042	######	0.0053080000000	#######	2.882879402024E-06
0.9879280765763	######	0.0048403000000	#######	3.204945741399E-08
0.9883150164971	######	0.0045276000000	#######	4.115696374842E-10
0.9882754333720	######	0.0042689000000	#######	1.930160453797E-12
0.9881185437487	######	0.0040310000000	#######	4.444238969120E-11
0.9879407601626	######	0.0038855000000	#######	5.272471927721E-12
0.9877168000000	######	0.0037454000000	#######	4.616623776617E-12
0.9874797586300	######	0.0036301000000	#######	1.091791064768E-11
0.9872943326378	######	0.0035367000000	#######	6.108345411770E-13
sens-x pol		EML-x pol		confinement -x pol

confinement-y pol	Total loss-x pol	Total loss-y pol
#DIV/0!	#DIV/0!	#DIV/0!
0.000000000000E+00	3.240198087887E-02	########
0.000000000000E+00	5.753191892311E-03	########
0.000000000000E+00	4.692213547945E-03	########
0.000000000000E+00	4.201127519642E-03	########
0.000000000000E+00	3.837702760544E-03	########
0.000000000000E+00	3.550300059360E-03	########
0.000000000000E+00	3.323900003485E-03	########
[redacted]		
#DIV/0!	#DIV/0!	#DIV/0!
#DIV/0!	#DIV/0!	#DIV/0!
#DIV/0!	#DIV/0!	#DIV/0!
#REF!	7.463296956207E-03	#REF!
#REF!	5.347926694919E-03	#REF!
#REF!	4.803879891078E-03	#REF!
#REF!	4.405302660416E-03	#REF!
#REF!	4.123800048332E-03	#REF!
0.000000000000E+00	3.871400002279E-03	########
0.000000000000E+00	3.666100001802E-03	########
0.000000000000E+00	3.500000004305E-03	########
0.000000000000E+00	3.359400006278E-03	########
#REF!	1.480400003409E-03	#REF!
#DIV/0!	#DIV/0!	#DIV/0!
#DIV/0!	#DIV/0!	#DIV/0!
0.000000000000E+00	6.331343787654E-03	########
0.000000000000E+00	5.310882879402E-03	########
0.000000000000E+00	4.840332049457E-03	########
0.000000000000E+00	4.527600411570E-03	########
0.000000000000E+00	4.268900001930E-03	########
0.000000000000E+00	4.031000044442E-03	########
0.000000000000E+00	3.885500005272E-03	########
0.000000000000E+00	3.745400004617E-03	########
0.000000000000E+00	3.630100010918E-03	########
0.000000000000E+00	3.536700000611E-03	########
	Total loss-x pol	

v para-x pol	NA- x pol	spot-x pol	nonlinear- x pol
#DIV/0!	#DIV/0!	#DIV/0!	#DIV/0!
1.51023034933994E+00	5.87099613299289E-01	1.976781793916E-04	1.061331864500E-05
1.56566298087681E+00	5.36021106573298E-01	1.872499639311E-04	1.405688856465E-05
1.60460429997260E+00	4.90388363514024E-01	1.809007484714E-04	1.753083985010E-05
1.63339741035646E+00	4.50421782295996E-01	1.766403098703E-04	2.103545851528E-05
1.65680495457256E+00	4.15548479660871E-01	1.734166547565E-04	2.456205844861E-05
1.67576078633849E+00	3.85076436860734E-01	1.709494360204E-04	2.810386756448E-05
1.69242262896977E+00	3.58356427137772E-01	1.688788748923E-04	3.165686746988E-05
[redacted]			
#DIV/0!	#DIV/0!	#DIV/0!	#DIV/0!
#DIV/0!	#DIV/0!	#DIV/0!	#DIV/0!
#DIV/0!	#DIV/0!	#DIV/0!	#DIV/0!
1.52934174261779E+00	5.30405863758698E-01	2.326529308348E-04	7.898860375502E-06
1.57535796683572E+00	4.77128524601461E-01	2.227210242897E-04	1.027728394696E-05
1.60772411876772E+00	4.31647223235765E-01	2.165067840849E-04	1.267841895004E-05
1.63261039493112E+00	3.92978693200072E-01	2.121027530101E-04	1.509477477477E-05
1.65241091244033E+00	3.59978302748698E-01	2.088077070551E-04	1.751945446280E-05
1.66768436367700E+00	3.31646516697976E-01	2.063827720916E-04	1.994907597536E-05
1.67948705005424E+00	3.07150319011451E-01	2.045743679366E-04	2.238115626265E-05
1.69062934956220E+00	2.85826381129099E-01	2.029170082837E-04	2.481585833110E-05
1.69777852519067E+00	2.67126129676905E-01	2.018781452489E-04	2.725089643168E-05
1.70674200487718E+00	2.50624253057648E-01	2.006018252612E-04	2.968731327953E-05
#DIV/0!	#DIV/0!	#DIV/0!	#DIV/0!
#DIV/0!	#DIV/0!	#DIV/0!	#DIV/0!
1.53630908527659E+00	5.05262220648463E-01	2.503123216375E-04	6.917091265900E-06
1.57936834787626E+00	4.51804596533595E-01	2.404121488416E-04	8.942903849625E-06
1.60868210705162E+00	4.06919189478281E-01	2.343591360431E-04	1.098738931505E-05
1.63093929782539E+00	3.69194671501851E-01	2.300881408170E-04	1.304329549140E-05
1.64757620223992E+00	3.37290677235121E-01	2.270624107542E-04	1.510617053093E-05
1.66115403101679E+00	3.10095961379719E-01	2.246915660750E-04	1.717384354853E-05
1.67322073425353E+00	2.86723308291740E-01	2.226548705048E-04	1.924544378698E-05
1.68135373659805E+00	2.66480786594051E-01	2.213178394018E-04	2.132222485207E-05
1.68598766997203E+00	2.48811455238845E-01	2.205684938823E-04	2.340410545494E-05
1.69272346322851E+00	2.33288690679414E-01	2.194949841229E-04	2.549423220502E-05
v para-x pol	NA- x pol	spot-x pol	nonlinear- x pol

	birefringence
#DIV/0!	0.00000000000000E+00
2.578381521338E+01	1.38560000000000E+00
2.302441463276E+01	1.43240000000000E+00
2.065903448215E+01	1.46300000000000E+00
1.866896203233E+01	1.48400000000000E+00
1.700984959834E+01	1.49890000000000E+00
1.560507221129E+01	1.50990000000000E+00
1.441430962385E+01	1.51820000000000E+00
[redacted]	
#DIV/0!	0.00000000000000E+00
#DIV/0!	0.00000000000000E+00
#DIV/0!	0.00000000000000E+00
2.231571932594E+01	1.39070000000000E+00
1.966154914831E+01	1.42530000000000E+00
1.748666505318E+01	1.44760000000000E+00
1.571587663115E+01	1.46270000000000E+00
1.425541105527E+01	1.47340000000000E+00
1.302610087143E+01	1.48130000000000E+00
1.197911397082E+01	1.48730000000000E+00
1.109396735678E+01	1.49190000000000E+00
1.031110275821E+01	1.49560000000000E+00
9.648644740482E+00	1.49850000000000E+00
#DIV/0!	0.00000000000000E+00
#DIV/0!	0.00000000000000E+00
2.088152270810E+01	1.39220000000000E+00
1.831468637194E+01	1.42230000000000E+00
1.622734553222E+01	1.44170000000000E+00
1.454153357286E+01	1.45480000000000E+00
1.315081229199E+01	1.46410000000000E+00
1.199672815617E+01	1.47090000000000E+00
1.103123122260E+01	1.47600000000000E+00
1.019177042184E+01	1.48000000000000E+00
9.453848722259E+00	1.48320000000000E+00
8.831824935986E+00	1.48570000000000E+00
	birefringence

[redacted]
