# Supplementary material for: Illegal drugs sensor: Performance evaluation and identification based on terahertz photonic crystal fiber
Source: PLoS One. 2025 Jun 27;20(6):e0327013. doi: 10.1371/journal.pone.0327013 (PMC12204572; doi:10.1371/journal.pone.0327013)
Supplement: S2 File — (PDF) [file pone.0327013.s002.pdf]

wave	freq	p	nr	l	aff	real-x pol	real-y p
#DIV/0!							
1.88E-04	1.60E+12	7.00E-05	1.562	0.000107231	0.95	1.41610000000000	
1.88E-04	1.60E+12	8.00E-05	1.562	0.00012255	0.95	1.44610000000000	
1.88E-04	1.60E+12	9.00E-05	1.562	0.000137869	0.95	1.46780000000000	
1.88E-04	1.60E+12	1.00E-04	1.562	0.000153188	0.95	1.48400000000000	
1.88E-04	1.60E+12	1.10E-04	1.562	0.000168506	0.95	1.49630000000000	
1.88E-04	1.60E+12	1.20E-04	1.562	0.000183825	0.95	1.50590000000000	
1.88E-04	1.60E+12	1.30E-04	1.562	0.000199144	0.95	1.51350000000000	
1.88E-04	1.60E+12	1.40E-04	1.562	0.000214463	0.95	1.51960000000000	
1.88E-04	1.60E+12	1.50E-04	1.562	0.000229781	0.95	1.52470000000000	
1.88E-04	1.60E+12	1.60E-04	1.562	0.0002451	0.95	1.52890000000000	
#DIV/0!							
#DIV/0!							
#DIV/0!							
1.88E-04	1.60E+12	7.00E-05	1.518	0.000107231	0.95	1.37500000000000	
1.88E-04	1.60E+12	8.00E-05	1.518	0.00012255	0.95	1.40410000000000	
1.88E-04	1.60E+12	9.00E-05	1.518	0.000137869	0.95	1.42530000000000	
1.88E-04	1.60E+12	1.00E-04	1.518	0.000153188	0.95	1.44110000000000	
1.88E-04	1.60E+12	1.10E-04	1.518	0.000168506	0.95	1.45320000000000	
1.88E-04	1.60E+12	1.20E-04	1.518	0.000183825	0.95	1.46270000000000	
1.88E-04	1.60E+12	1.30E-04	1.518	0.000199144	0.95	1.47020000000000	
1.88E-04	1.60E+12	1.40E-04	1.518	0.000214463	0.95	1.47630000000000	
1.88E-04	1.60E+12	1.50E-04	1.518	0.000229781	0.95	1.48130000000000	
1.88E-04	1.60E+12	1.60E-04	1.518	0.0002451	0.95	1.48550000000000	
#DIV/0!							
#DIV/0!							
1.88E-04	1.60E+12	7.00E-05	1.5022	0.000107231	0.95	1.36040000000000	
1.88E-04	1.60E+12	8.00E-05	1.5022	0.00012255	0.95	1.38920000000000	
1.88E-04	1.60E+12	9.00E-05	1.5022	0.000137869	0.95	1.41020000000000	
1.88E-04	1.60E+12	1.00E-04	1.5022	0.000153188	0.95	1.42590000000000	
1.88E-04	1.60E+12	1.10E-04	1.5022	0.000168506	0.95	1.43790000000000	
1.88E-04	1.60E+12	1.20E-04	1.5022	0.000183825	0.95	1.44730000000000	
1.88E-04	1.60E+12	1.30E-04	1.5022	0.000199144	0.95	1.45480000000000	
1.88E-04	1.60E+12	1.40E-04	1.5022	0.000214463	0.95	1.46090000000000	
1.88E-04	1.60E+12	1.50E-04	1.5022	0.000229781	0.95	1.46580000000000	
1.88E-04	1.60E+12	1.60E-04	1.5022	0.0002451	0.95	1.47000000000000	
wave	freq	p	nr	l	aff	real-x pol	

imag-x pol	imag-y p	area-x pol	area-y p	power-x pol	power-y p
					#######
9.8818000000000E-09		2.5025000000000E-08		8.9916000000000E-01	#######
3.1466000000000E-10		3.0665000000000E-08		9.2337000000000E-01	#######
1.1217000000000E-11		3.6987000000000E-08		9.3877000000000E-01	#######
4.3812000000000E-13		4.3968000000000E-08		9.4929000000000E-01	#######
1.8432000000000E-14		5.1604000000000E-08		9.5686000000000E-01	#######
8.4218000000000E-16		5.9891000000000E-08		9.6255000000000E-01	#######
2.8451000000000E-17		6.8824000000000E-08		9.6695000000000E-01	#######
2.0555000000000E-18		7.8406000000000E-08		9.7044000000000E-01	#######
2.1838000000000E-17		8.8630000000000E-08		9.7327000000000E-01	#######
1.0432000000000E-17		9.9495000000000E-08		9.7561000000000E-01	#######
					#######
					#######
					#######
3.6874000000000E-08		2.5817000000000E-08		8.8316000000000E-01	#######
1.4557000000000E-09		3.1539000000000E-08		9.1086000000000E-01	#######
6.4252000000000E-11		3.7966000000000E-08		9.2838000000000E-01	#######
3.1136000000000E-12		4.5069000000000E-08		9.4028000000000E-01	#######
1.6308000000000E-13		5.2838000000000E-08		9.4881000000000E-01	#######
9.1145000000000E-15		6.1272000000000E-08		9.5517000000000E-01	#######
5.5203000000000E-16		7.0370000000000E-08		9.6006000000000E-01	#######
2.3865000000000E-17		8.0130000000000E-08		9.6393000000000E-01	#######
3.3645000000000E-17		9.0552000000000E-08		9.6704000000000E-01	#######
3.1200000000000E-18		1.0164000000000E-07		9.6960000000000E-01	#######
					#######
					#######
5.9375000000000E-08		2.6125000000000E-08		8.7660000000000E-01	#######
2.5392000000000E-09		3.1874000000000E-08		9.0572000000000E-01	#######
1.2135000000000E-10		3.8337000000000E-08		9.2409000000000E-01	#######
6.3717000000000E-12		4.5479000000000E-08		9.3654000000000E-01	#######
3.6222000000000E-13		5.3291000000000E-08		9.4544000000000E-01	#######
2.2045000000000E-14		6.1768000000000E-08		9.5205000000000E-01	#######
1.4106000000000E-15		7.0909000000000E-08		9.5713000000000E-01	#######
1.0517000000000E-16		8.0713000000000E-08		9.6113000000000E-01	#######
7.7133000000000E-19		9.1177000000000E-08		9.6433000000000E-01	#######
1.2179000000000E-16		1.0230000000000E-07		9.6695000000000E-01	#######
imag x pole		area x pole		power-x pol	

sens-x pol	sens y-p	EML-x pol	EML-y p	confinement -x pol
#DIV/0!	######		#######	#DIV/0!
0.9917999576301	######	0.0056701000000	#######	2.876272166684E-03
0.9973749671530	######	0.0050932000000	#######	9.158734238385E-05
0.9990180814825	######	0.0045817000000	#######	3.264905674441E-06
0.9991852964960	######	0.0042010000000	#######	1.275225527401E-07
0.9988741027869	######	0.0039422000000	#######	5.364958669101E-09
0.9984083272462	######	0.0036581000000	#######	2.451313417938E-10
0.9979358440700	######	0.0034494000000	#######	8.281165315461E-12
0.9975172940247	######	0.0032890000000	#######	5.982895260599E-13
0.9970799108021	######	0.0031117000000	#######	6.356335037750E-12
0.9967315193930	######	0.0029692000000	#######	3.036417580081E-12
#DIV/0!	######		#######	#DIV/0!
#DIV/0!	######		#######	#DIV/0!
#DIV/0!	######		#######	#DIV/0!
0.9750086400000	######	0.0063591000000	#######	1.073282801456E-02
0.9847485791610	######	0.0057681000000	#######	4.237071579107E-04
0.9887608503473	#REF!	0.0053704000000	#######	1.870167775646E-05
0.9904552355839	#REF!	0.0049882000000	#######	9.062681918463E-07
0.9911186209744	#REF!	0.0046757000000	#######	4.746731009966E-08
0.9912819170028	#REF!	0.0044053000000	#######	2.652935969483E-09
0.9912740307441	#REF!	0.0042086000000	#######	1.606780671714E-10
0.9911574476732	######	0.0040336000000	#######	6.946329136181E-12
0.9909989333693	######	0.0038768000000	#######	9.792970617507E-12
0.9908130595759	#REF!	0.0037476000000	#######	9.081310247175E-13
#DIV/0!	######		#######	#DIV/0!
#DIV/0!	######		#######	#DIV/0!
0.9679715671861	######	0.0066556000000	#######	1.728214089507E-02
0.9793928764757	######	0.0060521000000	#######	7.390789416547E-04
0.9843766827400	######	0.0056747000000	#######	3.532105764406E-05
0.9866543151694	######	0.0052907000000	#######	1.854595657113E-06
0.9877181778983	######	0.0049786000000	#######	1.054305191581E-07
0.9881638291992	######	0.0047408000000	#######	6.416586038429E-09
0.9883150164971	######	0.0045276000000	#######	4.105800075213E-10
0.9883013799713	######	0.0043328000000	#######	3.061158329152E-11
0.9882770678128	######	0.0042161000000	#######	2.245091997742E-13
0.9881308095238	######	0.0040834000000	#######	3.544912740396E-11
sens-x pol		EML-x pol		confinement -x pol

confinement-y pol	Total loss-x pol	Total loss-y pol
#DIV/0!	#DIV/0!	#DIV/0!
0.000000000000E+00	8.546372166684E-03	########
0.000000000000E+00	5.184787342384E-03	########
0.000000000000E+00	4.584964905674E-03	########
0.000000000000E+00	4.201127522553E-03	########
0.000000000000E+00	3.942205364959E-03	########
0.000000000000E+00	3.658100245131E-03	########
0.000000000000E+00	3.449400008281E-03	########
0.000000000000E+00	3.289000000598E-03	########
0.000000000000E+00	3.111700006356E-03	########
0.000000000000E+00	2.969200003036E-03	########
#DIV/0!	#DIV/0!	#DIV/0!
#DIV/0!	#DIV/0!	#DIV/0!
#DIV/0!	#DIV/0!	#DIV/0!
0.000000000000E+00	1.709192801456E-02	########
0.000000000000E+00	6.191807157911E-03	########
#REF!	5.389101677756E-03	#REF!
#REF!	4.989106268192E-03	#REF!
#REF!	4.675747467310E-03	#REF!
#REF!	4.405302652936E-03	#REF!
#REF!	4.208600160678E-03	#REF!
0.000000000000E+00	4.033600006946E-03	########
0.000000000000E+00	3.876800009793E-03	########
#REF!	3.747600000908E-03	#REF!
#DIV/0!	#DIV/0!	#DIV/0!
#DIV/0!	#DIV/0!	#DIV/0!
0.000000000000E+00	2.393774089507E-02	########
0.000000000000E+00	6.791178941655E-03	########
0.000000000000E+00	5.710021057644E-03	########
0.000000000000E+00	5.292554595657E-03	########
0.000000000000E+00	4.978705430519E-03	########
0.000000000000E+00	4.740806416586E-03	########
0.000000000000E+00	4.527600410580E-03	########
0.000000000000E+00	4.332800030612E-03	########
0.000000000000E+00	4.216100000225E-03	########
0.000000000000E+00	4.083400035449E-03	########
	Total loss-x pol	

v para-x pol	NA- x pol	spot-x pol	nonlinear- x pol
#DIV/0!	#DIV/0!	#DIV/0!	#DIV/0!
1.54623299392586E+00	5.55876418591623E-01	1.334923208434E-04	3.695852307692E-05
1.58291447914564E+00	5.17069917105056E-01	1.474783210401E-04	3.016099918474E-05
1.61121892115835E+00	4.81951622348684E-01	1.619022450368E-04	2.500573282505E-05
1.63339741035646E+00	4.50421782295996E-01	1.766403098703E-04	2.103545851528E-05
1.65232328903232E+00	4.22148092977633E-01	1.914200791902E-04	1.792277807922E-05
1.66825674042711E+00	3.96777800473084E-01	2.062937825923E-04	1.544283848992E-05
1.68248692982507E+00	3.73973930391859E-01	2.211337674769E-04	1.343843775427E-05
1.69581592209595E+00	3.53411087498165E-01	2.358550421320E-04	1.179612580670E-05
1.70558158286979E+00	3.34823426200571E-01	2.509568101125E-04	1.043537222160E-05
1.71496818887323E+00	3.17965757954755E-01	2.659404742842E-04	9.295814262023E-06
#DIV/0!	#DIV/0!	#DIV/0!	#DIV/0!
#DIV/0!	#DIV/0!	#DIV/0!	#DIV/0!
#DIV/0!	#DIV/0!	#DIV/0!	#DIV/0!
1.50875904752831E+00	5.49896083834598E-01	1.385863704829E-04	3.582472944184E-05
1.54660353334567E+00	5.11754463272126E-01	1.525084229848E-04	2.932518596024E-05
1.57535796683572E+00	4.77128221279333E-01	1.670407682173E-04	2.436092925249E-05
1.59853666791694E+00	4.45992626456462E-01	1.818434891131E-04	2.052157891233E-05
1.61743092747477E+00	4.18060345320547E-01	1.968564926856E-04	1.750420227866E-05
1.63261039493112E+00	3.92978693200072E-01	2.121027530101E-04	1.509477477477E-05
1.64642420845525E+00	3.70412590123781E-01	2.272675539467E-04	1.314320079579E-05
1.65776638921780E+00	3.50059247584646E-01	2.426039397346E-04	1.154233171097E-05
1.66768436367700E+00	3.31645803645773E-01	2.579784651145E-04	1.021387755102E-05
1.67515536717163E+00	3.14928862294953E-01	2.736421729749E-04	9.099636363636E-06
#DIV/0!	#DIV/0!	#DIV/0!	#DIV/0!
#DIV/0!	#DIV/0!	#DIV/0!	#DIV/0!
1.49450062422376E+00	5.47621979536197E-01	1.406907126858E-04	3.540237473684E-05
1.53236741703534E+00	5.09760103297614E-01	1.546372190249E-04	2.901697433645E-05
1.56113890244665E+00	4.75337830969910E-01	1.692324030083E-04	2.412518037405E-05
1.58392580074972E+00	4.44376336056977E-01	1.841823115688E-04	2.033657380329E-05
1.60272434764314E+00	4.16589264783006E-01	1.993102067179E-04	1.735540785498E-05
1.61815913905441E+00	3.91640605834116E-01	2.146228505928E-04	1.497356300997E-05
1.63093929782539E+00	3.69194671501851E-01	2.300881408170E-04	1.304329549140E-05
1.64117853282918E+00	3.48947162049374E-01	2.457643915648E-04	1.145896001883E-05
1.65216535515670E+00	3.30631830138355E-01	2.610593785119E-04	1.014386347434E-05
1.65869503732695E+00	3.14011798668063E-01	2.770641303111E-04	9.040929032258E-06

v para-x pol | NA- x pol | spot-x pol | nonlinear- x pol

	birefringence
#DIV/0!	0.00000000000000E+00
2.408884262240E+01	1.41610000000000E+00
2.203262287085E+01	1.44610000000000E+00
2.023564513001E+01	1.46780000000000E+00
1.866896203233E+01	1.48400000000000E+00
1.731694249438E+01	1.49630000000000E+00
1.613567551353E+01	1.50590000000000E+00
1.510394828944E+01	1.51350000000000E+00
1.420052922028E+01	1.51960000000000E+00
1.337763138099E+01	1.52470000000000E+00
1.264884337243E+01	1.52890000000000E+00
#DIV/0!	0.00000000000000E+00
#DIV/0!	0.00000000000000E+00
#DIV/0!	0.00000000000000E+00
2.329933297459E+01	1.37500000000000E+00
2.137244729442E+01	1.40410000000000E+00
1.966154914831E+01	1.42530000000000E+00
1.817034269712E+01	1.44110000000000E+00
1.686621801230E+01	1.45320000000000E+00
1.571587663115E+01	1.46270000000000E+00
1.471422023673E+01	1.47020000000000E+00
1.382084053979E+01	1.47630000000000E+00
1.302610087143E+01	1.48130000000000E+00
1.230383318113E+01	1.48550000000000E+00
#DIV/0!	0.00000000000000E+00
#DIV/0!	0.00000000000000E+00
2.298728081684E+01	1.36040000000000E+00
2.110428081907E+01	1.38920000000000E+00
1.942610351452E+01	1.41020000000000E+00
1.795450500216E+01	1.42590000000000E+00
1.667016424249E+01	1.43790000000000E+00
1.554030809343E+01	1.44730000000000E+00
1.454153357286E+01	1.45480000000000E+00
1.364979856810E+01	1.46090000000000E+00
1.287752628181E+01	1.46580000000000E+00
1.215641779414E+01	1.47000000000000E+00
	birefringence
